# Activated Platelets and Platelet-Derived Extracellular Vesicles Mediate COVID-19-Associated Immunothrombosis

**DOI:** 10.3389/fcell.2022.914891

**Published:** 2022-07-06

**Authors:** Marie Ebeyer-Masotta, Tanja Eichhorn, René Weiss, Lucia Lauková, Viktoria Weber

**Affiliations:** Center for Biomedical Technology, Department for Biomedical Research, University for Continuing Education Krems, Krems, Austria

**Keywords:** apheresis, coagulopathy, COVID-19, extracellular vesicles, heparin, immunothrombosis, platelets, platelet factor 4

## Abstract

Activated platelets and platelet-derived extracellular vesicles (EVs) have emerged as central players in thromboembolic complications associated with severe coronavirus disease 2019 (COVID-19). Platelets bridge hemostatic, inflammatory, and immune responses by their ability to sense pathogens via various pattern recognition receptors, and they respond to infection through a diverse repertoire of mechanisms. Dysregulated platelet activation, however, can lead to immunothrombosis, a simultaneous overactivation of blood coagulation and the innate immune response. Mediators released by activated platelets in response to infection, such as antimicrobial peptides, high mobility group box 1 protein, platelet factor 4 (PF4), and PF4^+^ extracellular vesicles promote neutrophil activation, resulting in the release of neutrophil extracellular traps and histones. Many of the factors released during platelet and neutrophil activation are positively charged and interact with endogenous heparan sulfate or exogenously administered heparin via electrostatic interactions or via specific binding sites. Here, we review the current state of knowledge regarding the involvement of platelets and platelet-derived EVs in the pathogenesis of immunothrombosis, and we discuss the potential of extracorporeal therapies using adsorbents functionalized with heparin to deplete platelet-derived and neutrophil-derived mediators of immunothrombosis.

## Introduction

Next to the lung inflammatory syndrome, thrombotic events and endothelial dysfunction are key pathogenic mechanisms of severe coronavirus disease-19 (COVID-19) caused by the severe acute respiratory syndrome coronavirus 2 (SARS-CoV-2) (Bonaventura et al., 2021; Hottz et al., 2020; Klok et al., 2020; Middeldorp et al., 2020; Middleton et al., 2020; Tang N. et al., 2020). Here, we focus on the involvement of platelets and platelet-derived extracellular vesicles (pEVs) in COVID-19-related immunothrombosis. We discuss the interaction of heparan sulfate and heparin with activated platelets and with mediators of immunothrombosis and highlight potential therapeutic implications of this interaction.

## Immunothrombosis as a Hallmark of COVID-19

SARS-CoV-2 is an enveloped, single-stranded RNA virus (Masters, 2006; Zhou et al., 2020; Hu et al., 2021). Binding to its target cells depends mainly on the interaction of the viral spike protein with angiotensin-converting enzyme 2 (ACE2) on the surface of its target cells.

The clinical spectrum of COVID-19 ranges from asymptomatic or mild forms to severe disease requiring intensive care (Wiersinga et al., 2020). COVID-19 was first described as a pulmonary condition (Zhou et al., 2020; Zhu et al., 2020), but can be associated with gastrointestinal, cardiovascular, renal, or neurological dysfunctions alike (Gupta et al., 2020; Renu et al., 2020). Severe SARS-CoV-2 infection frequently induces a prothrombotic state, which can progress to multiorgan failure (Yang et al., 2020; Lopes-Pacheco et al., 2021). Laboratory parameters associated with a higher thrombotic risk in COVID-19 patients include elevated D-dimer levels, low fibrinogen, and low lymphocyte counts (Thomas and Scully, 2022).

**FIGURE 1:**
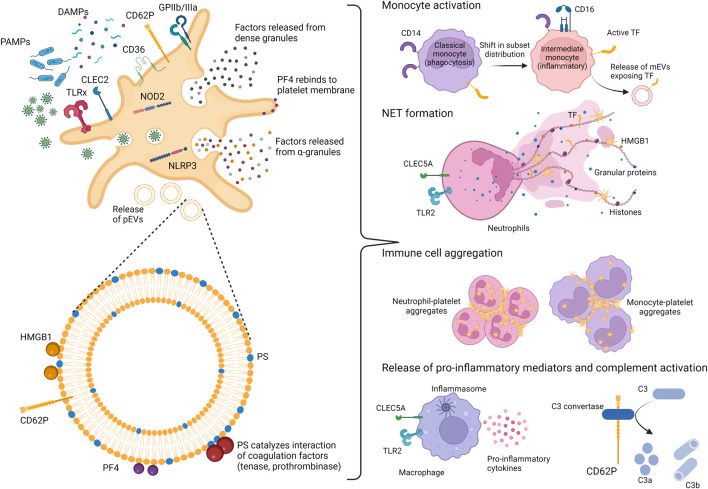
Platelets and platelet-derived EVs as mediators of immunothrombosis. Platelets act as sentinels by expressing pathogen-recognition receptors (PRRs), able to recognize pathogen-associated molecular patterns (PAMPs) or damage-associated molecular patterns (DAMPs). Upon activation, platelets expose on their surface activation markers (CD62P and GPIIb/IIIa) and release various mediators from their α-and dense-granules, including PF4 and HMGB1, both exerting affinity for heparin. Activated platelets also shed platelet-derived extracellular vesicles (pEVs), which expose phosphatidylserine (PS) as well as PF4 and HMGB1. Altogether, platelet activation and pEV release induce numerous processes supporting immunothrombosis, namely a shift in monocyte subsets towards inflammatory monocytes, the stimulation of neutrophil extracellular trap (NET) formation and enhanced interaction of platelets with other platelets, as well as with monocytes or neutrophils. TLR, Toll-like receptor; CLEC2, C-type lectin receptor 2; CLEC5A, C-type lectin receptor 5A; NOD2, nucleotide-binding oligomerization domain 2; NLRP3, nucleotide-binding domain rich leucine repeat containing protein; GP, glycoprotein; TF, tissue factor; PF4, platelet factor 4; HMGB1, high mobility group box 1 protein; mEVs, monocyte-derived EVs; C3, complement component 3.

The thrombotic events associated with severe COVID-19 are best described as immunothrombosis, a simultaneous overactivation of coagulation and the innate immune system (Engelmann and Massberg, 2013; Ackermann et al., 2020; Connors and Levy, 2020; Skendros et al., 2020). During immunothrombosis, deregulated complement activation enhances neutrophil activation and recruitment to the infected lungs, and promotes tissue factor (TF) expression, resulting in microvascular thrombosis and endothelial dysfunction (Bonaventura et al., 2021). The excessive release of neutrophil extracellular traps (NETs), which consist in DNA fibers associated with neutrophil elastase, antimicrobial peptides, TF citrullinated histone H3 (Nomura et al., 2019) as well as with high-mobility group box 1 protein (HMGB1) (Garcia-Romo et al., 2011) and activated platelets (Brinkmann et al., 2004; Li et al., 2020), further promotes thrombosis and tissue damage (Middleton et al., 2020). In addition to this complement/neutrophil/TF axis, platelets are centrally involved in initiating and propagating immunothrombosis in COVID-19 (Foley and Conway, 2016; Martinod and Deppermann, 2021), as discussed in detail below and shown in Figure 1. Altogether, the disproportionate activation of the innate immune response after infection with SARS-CoV-2 leads to an excessive release of inflammatory mediators (Fara et al., 2020; Huang et al., 2020), giving rise to a tissue-damaging environment (Elrobaa and New, 2021) and uncontrolled microthrombus formation (Helms et al., 2020; Iba et al., 2020; McFadyen et al., 2020).

## Platelets as Mediators of Immunothrombosis

### Platelets Are Sentinels of Endogenous and Exogenous Signals of Infection

Platelets are small (1–3 µm), short-lived (up to 9 days), anucleate entities derived from megakaryocytes residing in the bone marrow. With a concentration of 150,000 to 400,000 platelets per µL, they represent the second most abundant formed elements of blood (Ghoshal and Bhattacharyya, 2014). They are equipped with an array of surface and intracellular receptors to detect pathogens, enabling their function as intravascular sentinels. Platelets can contribute to the propagation of inflammatory processes through the release of mediators from their dense- and α-granules and through their interaction with other immune cells (Morrell et al., 2014).

SARS-CoV-2 can trigger an innate immune response via pathogen-associated molecular patterns (PAMPs) such as single-stranded RNA or the spike protein (Khan et al., 2021), and it stimulates the release of damage-associated molecular patterns (DAMPs) including HMGB1 (Chen et al., 2020). Platelets express with a wide range of pattern recognition receptors (PRRs) to sense PAMPs and DAMPs during viral infection. Human platelet PRRs include Toll-like receptors (TLRs) 1–10, which have all been detected in human platelets using RT-PCR (Koupenova et al., 2015). The presence of TLR1 (Shiraki et al., 2004), TLR2 (Cognasse et al., 2005; Aslam et al., 2006; Claushuis et al., 2019; Marín Oyarzún et al., 2020), TLR3 (Anabel et al., 2014), TLR4 (Cognasse et al., 2005; Claushuis et al., 2019; Marín Oyarzún et al., 2020), TLR5 (Claushuis et al., 2019), TLR6 (Shiraki et al., 2004; Aslam et al., 2006), TLR7 (Koupenova et al., 2014), TLR8 (Leroy et al., 2019), TLR9 (Cognasse et al., 2005; Aslam et al., 2006; Thon et al., 2012; Claushuis et al., 2019) in human platelets has been confirmed using Western blotting and/or flow cytometry.

Platelets have been shown to endocytose RNA viruses (Seyoum et al., 2018) and to recognize viral RNA via their endosomal TLR3, TLR7 and TLR8 (Hottz et al., 2018; Azkur et al., 2020). Viral recognition by platelet TLRs elicits signaling through the adaptor molecule MyD88, resulting in platelet activation, aggregation, granule secretion and interleukin (IL)-1 beta release (D’Atri and Schattner, 2017; Marín Oyarzún et al., 2020). Notably, the secretory profile of immuno-activated platelets appears to be distinct from the secretory profile during hemostatic platelet activation (Banerjee et al., 2020). While platelet activation mediated by G-protein-coupled receptors occurs rapidly, platelet activation mediated by PRRs in response to infection can be delayed and sustained (Vieira-de-Abreu et al., 2012).

In addition to TLRs, platelets express nucleotide-binding oligomerization domain (NOD)-like receptors (NLRs), such as NOD2 which recognizes the bacterial component muramyl dipeptide (Zhang et al., 2015; Domínguez-Martínez et al., 2018) and viral RNA after internalization of SARS-CoV-2 by platelets. NOD2 acts via the mitochondrial antiviral signaling protein, a key mediator of the innate immune response to RNA viral infection (Vazquez and Horner, 2015; Fard et al., 2021), triggering the release of inflammatory cytokines.

Platelets also express the nucleotide-binding domain leucine rich repeat containing protein (NLRP3) (Hottz et al., 2015, 2013), a major sensor for the activation of the inflammasome by bacterial, viral or tissue damage signals.

In addition, they express C-type lectin receptor 2 (CLEC2) (Sung et al., 2019), which can recognize viral surface glycan patterns, as previously shown for human immunodeficiency virus (Chaipan et al., 2006) and dengue virus (Sung et al., 2019). CLEC2 might also interact with sialylated O-glycans of SARS-CoV-2 spike proteins (Gadanec et al., 2021), resulting in tyrosine-kinase-associated signal transduction and platelet activation (Meng et al., 2021).

Platelets can thus sense and respond to SARS-CoV-2 infection via different PRRs. There is no clear consensus, however, whether human platelets express the ACE2 on their surface, which has been identified as one of the main entry routes for SARS-CoV-2 into host cells (Lan et al., 2020). Some groups have described ACE2 expression on platelets (Zhang et al., 2020; Koupenova et al., 2021), while others have been unable to confirm its presence (Manne et al., 2020; Song et al., 2020; Zaid et al., 2020; Campbell et al., 2021a; Li et al., 2022). Platelets may also use other receptors to interact with SARS-CoV-2, including CD147 as well as glucose regulated protein 78 and kringle containing transmembrane protein 1 (Shen et al., 2021).

### Platelet Activation in COVID-19

Following pathogen recognition and activation, platelets mediate and propagate the innate immune response by various mechanisms, such as the release of chemokines including platelet factor 4 (PF4), modulation of leukocyte migration (Pan et al., 2015; Chimen et al., 2020; Middleton et al., 2020), leukocyte recruitment to thrombi (Swystun and Liaw, 2016), induction of NET formation (Clark et al., 2007), monocyte expression of TF (Lindmark et al., 2000; Ivanov et al., 2019) as well as the release of extracellular vesicles (EVs) (Suades et al., 2022). In addition, platelets sustain inflammation via the release of mediators from their α- and dense granules (D’Atri and Schattner, 2017), triggering the release of pro-inflammatory cytokines and inflammasome assembly by neutrophils and macrophages (Chen et al., 2017), complement activation (del Conde et al., 2005a), and exposure of CD40L (Lievens et al., 2010) leading to endothelial activation (Cognasse et al., 2022).

PF4 is an abundant platelet α-granule chemokine released during platelet activation. In addition to its soluble form, PF4 is displayed on the surface of activated platelets and of pEVs. PF4 expression is strongly elevated following trauma as well as in sepsis (Maharaj and Chang, 2018; Wegrzyn et al., 2021) and COVID-19 (Comer et al., 2021). Due to its positive charge at physiological pH, PF4 binds to endogenous heparan sulfate and other glycosaminoglycans as well as to exogenously administered heparin with high affinity, thereby promoting blood coagulation (Kowalska et al., 2010). By neutralizing the negatively charged heparan sulfate side chains of glycosaminoglycans on the surface of platelets and endothelial cells, PF4 facilitates platelet aggregation and thrombus formation. Its affinity for heparan sulfate induces the re-binding of soluble PF4 to the surface of platelets, pEVs, and monocytes (George and Onofre, 1982; Witt and Lander, 1994; Rauova et al., 2006, 2010). Beyond its hemostatic activity, PF4 is responsible for neutrophil recruitment to sites of inflammation/infection and strongly induces the formation of NETs, which are central mediators of COVID-19-associated coagulopathy (Middleton et al., 2020; Zuo et al., 2020).

PF4 shares its affinity for glycosaminoglycans with HMGB1, which is expressed and released by activated platelets (Rouhiainen et al., 2000; Maugeri et al., 2012; Vogel et al., 2015). HMGB1 interacts with heparin via two positively charged domains, box A and box B (Martinotti et al., 2015), and via its heparin-binding domain (Xu et al., 2011). HMGB1 plasma levels are elevated in conditions associated with abnormal coagulation, including sepsis (Eichhorn et al., 2021) and COVID-19, where HMGB1 has been correlated with disease severity (Chen et al., 2020). The biological functions of HMGB1 resemble those of activated platelets, including the induction of DNA externalization in neutrophils (Maugeri et al., 2014; Hoste et al., 2019) and microvascular thrombosis (Ito et al., 2007). HMGB1 signals through agonist receptors, such as the receptor for advanced glycation end products (RAGE) as well as other PRRs, including TLR2, TLR4 and TLR9 (Park et al., 2006, 2004). There is evidence that HMGB1-induced NET formation depends on the integrity of RAGE. Interaction of HMGB1 with heparin induces a conformational change and decreases its affinity for RAGE (Ling et al., 2011), abrogating the ability of activated platelets to elicit NET formation (Maugeri et al., 2014).

Beyond elevated levels of PF4 and HMGB1, patients suffering from severe COVID-19 display elevated markers of platelet activation including thromboxane A2 (Hottz et al., 2020; Zhang et al., 2020), surface expressed P-selectin (CD62P) and CD63 (Hottz et al., 2020; Manne et al., 2020; Nicolai et al., 2020; Taus et al., 2020; Zhang et al., 2020), activated glycoprotein (GP) IIb/IIIa (Bongiovanni et al., 2021; Léopold et al., 2021), as compared to healthy controls or to patients suffering from other pulmonary infections. Variable and partially conflicting results regarding the expression of individual platelet activation markers obtained in different studies may partly be due to the heterogeneity of COVID-19 patients. Furthermore, studies investigating single time points of platelet activation can merely provide snapshots of a highly dynamic process. As an example, increased platelet-monocyte aggregate formation has been described to trigger TF expression and immunothrombosis in critically ill COVID-19 patients (Hottz et al., 2020; Manne et al., 2020; Zaid et al., 2020), whereas other studies found diminished levels of circulating platelet-leukocyte aggregates in fatally ill patients, linked to a hypo-responsive platelet phenotype with impaired GPIIb/IIIa activation (Schrottmaier et al., 2021).

## Platelet-Derived EVs in Inflammation and Coagulation

EVs are membrane-enclosed vesicles released by almost all cell types and present in all body fluids (Lötvall et al., 2014; Yáñez-Mó et al., 2015). Platelet-derived EVs are the most abundant EV subset in the circulation (Arraud et al., 2014; Berckmans et al., 2019), and their release is enhanced under pathological conditions, including sepsis (Raeven et al., 2018) and COVID-19 (Cappellano et al., 2021; Krishnamachary et al., 2021; Traby et al., 2022). They are most commonly characterized in blood or plasma samples using flow cytometry. Typically, phosphatidylserine exposed on EVs is detected by staining with Annexin 5 or lactadherin, while CD41 is used as a marker for platelet origin (Weiss et al., 2018; Tripisciano et al., 2020).

Platelet activation mediated by PRRs as described above, or through platelet agonists, such as thrombin, ADP, or collagen triggers the release of pEVs (Heijnen et al., 1999; Taus et al., 2019). The release of pEVs from plasma membrane is associated with actin cytoskeletal rearrangement and depends on GPIIb/IIIa (Heinzmann et al., 2020). EV release alters the phospholipid composition of the plasma membrane monolayers, leading to an exposure of phosphatidylserine on the outer membrane of the EVs (Ståhl et al., 2019).

It is well established that the properties of pEVs depend on the agonists triggering their release and on their environment. Activation of platelets with ADP, thrombin, collagen, or with a combination of thrombin and collagen induces different responses in terms of surface protein patterns and EV cargo (Milioli et al., 2015), entailing functional differences of the resulting EV populations. As an example, pEVs enriched from platelet-rich plasma under physiological conditions support tissue regeneration (Wu et al., 2021), while pEVs released under pathological conditions sustain coagulation and immunothrombosis (Zaid and Merhi, 2022).

### Platelet-Derived EVs Provide a Large Pro-coagulant Surface

The exposure of phosphatidylserine on EVs results in the formation of a pro-coagulant surface (Nieuwland et al., 2000; Freyssinet and Toti, 2010). In fact, it has been estimated that the pro-coagulant activity of EVs is 50–100 fold higher than that of platelets (Sinauridze et al., 2007). Phosphatidylserine forms a catalytic, negatively charged surface, facilitating the formation of the tenase (factors VIIIa, IXa) and prothrombinase (factors Va, Xa) complexes of the coagulation cascade (Furie and Furie, 1992; Tripisciano et al., 2020). Beyond their ability to propagate coagulation by exposing phosphatidylserine, EVs may be initiators of coagulation via their exposure of TF. This is well-established for EVs derived from activated monocytes or endothelial cells (Nieuwland et al., 2000; Tripisciano et al., 2017; Hell et al., 2021), whereas TF expression on activated platelets and pEVs has long been controversial (Zillmann et al., 2001; Siddiqui et al., 2002; Panes et al., 2007; Bouchard et al., 2010, 2012; Camera et al., 2012; Østerud and Bouchard, 2019). Our own data do not support TF exposure on EVs released upon activation of platelets from medical grade platelet concentrates *in vitro* (Tripisciano et al., 2017). Still, TF might be transferred from monocytes to platelets *in vivo* in settings of inflammation. We and others have previously shown that pEVs shed from activated platelets preferentially bind to monocytes (Fendl et al., 2018; Weiss et al., 2018; Chimen et al., 2020) in the circulation. This binding is mediated by the interaction of P-selectin on pEVs and P-selectin glycoprotein ligand-1 (PSGL-1) on monocytes (Chimen et al., 2020). The P-selectin/PSGL-1 interaction enhances the exposure of TF on monocytes (Ivanov et al., 2019) and induces the release of TF-bearing monocyte EVs that can bind to activated platelets via P-selectin/PSGL-1 interaction (del Conde et al., 2005b). Increased EV-TF activity associated with severe COVID-19 has consistently been described by several groups (Hottz et al., 2020; Campbell et al., 2021b; Guervilly et al., 2021; Krishnamachary et al., 2021; Rosell et al., 2021). The assays used in these studies, however, did not further differentiate the cellular origin of the TF-exposing EVs.

### Platelet-Derived EVs Expose and Release Mediators of Immunothrombosis

Next to their pro-coagulant surface, pEVs expose and release mediators supporting immunothrombosis (Puhm et al., 2020). Like platelets, pEVs carry PF4 and HMGB1 on their surface, partially due to re-binding of soluble PF4 and HMGB1 to the pEV surface. Elevated levels of PF4^+^ pEVs have been reported in sepsis (Sartori et al., 2020), and there is evidence that HMGB1^+^ pEVs are significantly elevated in COVID-19 (Maugeri et al., 2022). In addition, oxidation-specific epitopes generated by lipid peroxidation in settings of inflammation and cell death (Miller et al., 2011; Weismann and Binder, 2012; Binder et al., 2016) have been identified on EVs (Tsiantoulas et al., 2015) and can further enhance immunothrombosis by acting as DAMPs.

### Platelet-Derived EVs Support NET Formation and Influence Other Immune Cells

Given that PF4 (Carestia et al., 2016) and HMGB1 (Denning et al., 2019) induce NET formation, PF4^+^ and HMGB1^+^ pEVs contribute to NET-associated coagulopathy. Moreover, pEVs have been directly associated with induction of NETosis in viral infection. Interaction of dengue virus with platelet CLEC2 was shown to trigger the release of pEVs, activating neutrophils through heterocomplexes of TLR2 and CLEC5A (Sung et al., 2019). EVs released from platelets after CLEC2 activation during SARS-CoV-2 infection might enhance neutrophil activation by a similar mechanism (Sung and Hsieh, 2021, 2019).

Furthermore, pEVs can propagate and spread platelet activation via the interaction of phosphatidylserine on EVs and CD36 exposed on platelets (Nergiz-Unal et al., 2011). As a scavenger receptor, CD36 recognizes oxidized phospholipids and lipoproteins, and participates in the internalization of apoptotic cells, certain bacterial and fungal pathogens, as well as modified low-density lipoproteins, and there is evidence that signaling following the interaction of phosphatidylserine and CD36 mediates a prothrombotic phenotype in platelets (Ghosh et al., 2008; Ramakrishnan et al., 2016).

Studies from our own group and others have further indicated that activated platelets and pEVs can shift the distribution of monocyte subsets towards intermediate CD14^+^CD16^+^ monocytes, which possess inflammatory characteristics (Passacquale et al., 2011; Fendl et al., 2019, 2021; Lee et al., 2021). The CD16 induction on monocytes appears to be triggered by platelet-derived transforming growth factor-beta and monocyte derived IL-6, suggesting an important role of activated platelets and pEVs in modulating phenotypical and functional features of human monocytes.

### Platelet-Derived EVs May Support Viral Propagation

There are indications that pEVs can propagate infection through the delivery of functional viral RNA from cell to cell (Valadi et al., 2007), which has already been described for several viruses (Bello-Morales et al., 2020; Nomura et al., 2020). Since SARS-CoV-2 RNA has been detected by reverse transcription-droplet digital polymerase chain reaction in exosomes isolated from plasma from COVID-19 patients (Barberis et al., 2021), it is conceivable that EVs might be involved in viral spreading.

## Therapeutic Potential of Platelet-Derived EV Depletion During COVID-19

Extracorporeal blood purification has been proposed as a supportive measure for the treatment of COVID-19 (Rock et al., 2021; Ronco et al., 2021). Given the central role of immunothrombosis, removing mediators of excessive cellular activation from the circulation may indeed be beneficial (Morris et al., 2021; Tang L. et al., 2020).

Potential extracorporeal approaches include plasma exchange (Rock et al., 2021), depletion of cytokines using polystyrene based adsorbents (Nassiri et al., 2021; Ruiz-Rodríguez et al., 2021), as well as hemadsorption using beads functionalized with heparin. The Seraph-100 Microbind Affinity Blood Filter consists of ultra-high molecular weight polyethylene beads with endpoint-attached heparin (Seffer et al., 2021). It has been developed following a biomimetic approach with the assumption that bacterial as well as viral pathogens bind to immobilized heparin in the same way as they interact with cellular heparan sulfate. In fact, heparan sulfate is an essential cofactor in SARS-CoV-2 infection, as it modifies the conformation of the spike protein to facilitate its recognition by ACE2 (Clausen et al., 2020).

So far, Seraph-100 has been exploited for its ability to deplete carbapenem-resistant *Enterobacteriaceae in vitro*, where it yielded promising results (McCrea et al., 2014). First clinical case reports on the capacity of the Seraph-100 Microbind Affinity Blood Filter to eliminate *Staphylococcus aureus* from the circulation have been published recently (Seffer et al., 2020). There is evidence that the viral load is associated with increased disease severity and mortality in COVID-19 (Fajnzylber et al., 2020), and that heparan sulfate is a co-factor for viral entry, as discussed further above (Kalra and Kandimalla, 2021; Tandon et al., 2021). It was therefore obvious to consider the application of Seraph-100 as a supportive therapy in COVID-19. The Seraph-100 Microbind Affinity Blood Filter obtained emergency use authorization for COVID-19 by the FDA in 2020, followed by a case series assessing its use in SARS-CoV-2 infected patients early in critical illness (Rifkin and Stewart, 2022). This study however did not collect data on virus elimination from the circulation. A follow-up study provided evidence that treatment with Seraph-100 decreased the SARS-CoV-2 nucleocapsid protein in critically ill patients (Kielstein et al., 2021), while effects on clinically relevant outcome parameters remain to be further assessed. A recently published interim analysis of a multicenter observational study in 12 hospitals monitoring 102 treatment sessions in 82 patients confirmed that the treatment was well tolerated. Mortality was correlated with late initiation of the treatment as well as with bacterial super-infection (Schmidt et al., 2022). Although the efficacy of this approach has yet to be consistently demonstrated, current data suggest that it can be deployed as an adjunct measure until directed pharmacologic countermeasures are available (Chitty et al., 2022).

**FIGURE 2:**
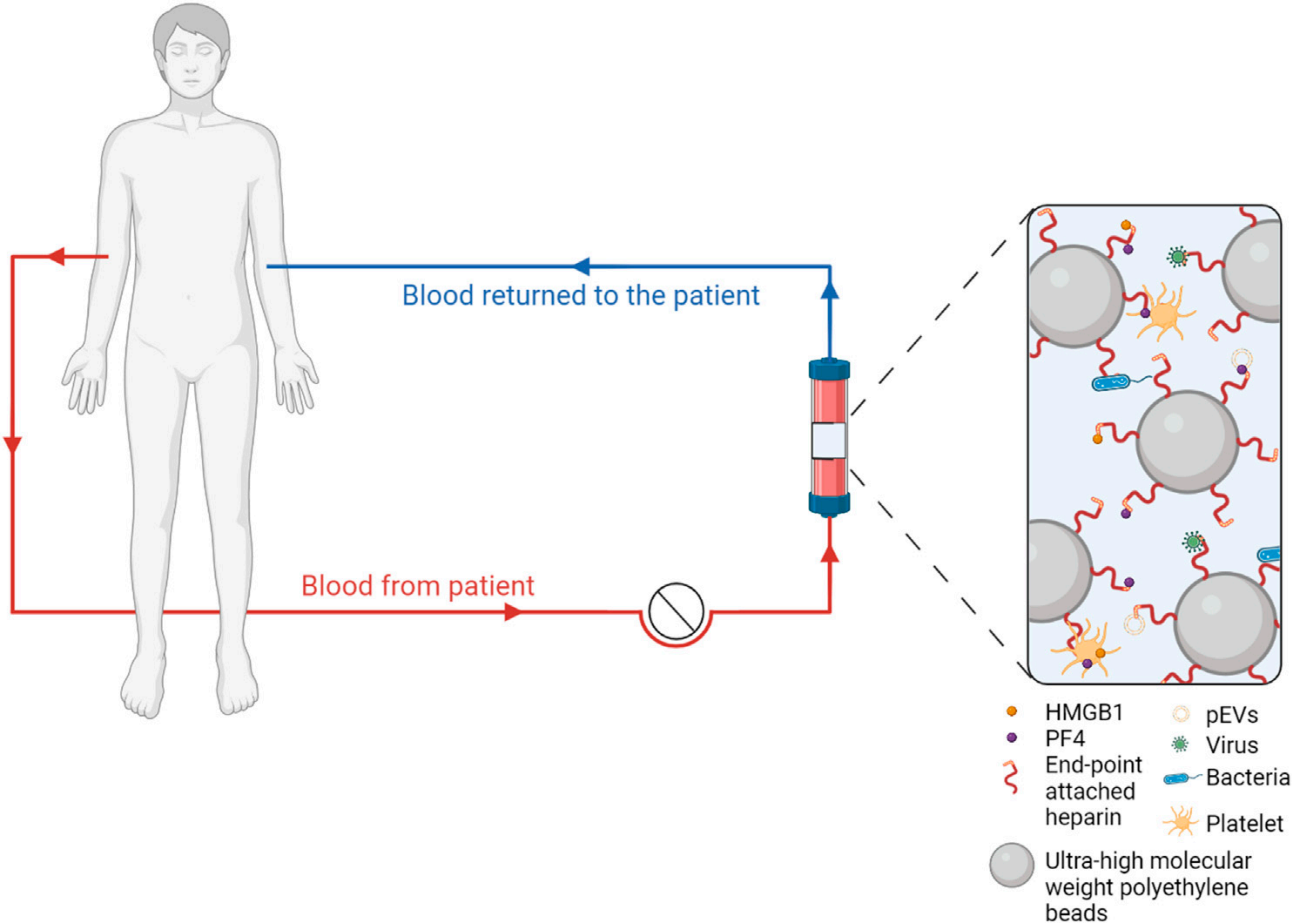
Extracorporeal blood purification with heparin-functionalized adsorbents. Human whole blood is recirculated over adsorbent columns containing beads functionalized with endpoint-attached heparin, which binds pathogens as well as mediators of immunothrombosis, including activated platelets, platelet-derived extracellular vesicles (EVs), platelet factor 4 (PF4) and high mobility group box 1 protein (HMGB1).

While this extracorporeal approach is currently mainly explored regarding pathogen depletion, we have provided evidence that heparin-immobilized adsorbents may exert beneficial effects by binding and depleting mediators of immunothrombosis, including activated platelets and PF4^+^ pEVs, as well as HMGB1 and histones/nucleosomes (Ebeyer-Masotta et al., 2022), as suggested in Figure 2, and we are currently assessing the ability of heparin-functionalized adsorbents to deplete NETs.

## Conclusion

Platelets are sentinels of viral infection and can propagate immunothrombosis at multiple levels, e.g., by the release or exposure of mediators, by providing pro-coagulant surfaces, by inducing a shift towards inflammatory monocyte subsets, and by contributing to viral spread via EVs. Activated platelets and pEVs are markers and central mediators of immunothrombosis in COVID-19 (Puhm et al., 2021). Adverse outcome in COVID-19 patients appears to be linked to increased basal platelet activation and diminished platelet reactivity, which aggravates over the course of the disease.

Both, cellular heparan sulfate and exogenous heparin interact with activated platelets, pEVs, and with various mediators of immunothrombosis in many ways, and thus both, extracorporeal therapy with immobilized heparin and administration of heparin may provide approaches to alleviate excessive immunothrombosis.
